# Leukaemia Inhibitory Factor Stimulates Proliferation of Olfactory Neuronal Progenitors via Inducible Nitric Oxide Synthase

**DOI:** 10.1371/journal.pone.0045018

**Published:** 2012-09-14

**Authors:** Estefania Lopez-Arenas, Alan Mackay-Sim, Juan Bacigalupo, Lorena Sulz

**Affiliations:** 1 Department of Biology, Faculty of Sciences, University of Chile, Santiago, Chile; 2 Millennium Institute for Cell Dynamics and Biotechnology, University of Chile, Santiago, Chile; 3 National Centre for Adult Stem Cell Research, Eskitis Institute for Cell and Molecular Therapies, Griffith University, Brisbane, QLD, Australia; 4 Laboratory of Embryology, School of Medicine, Faculty of Medical Sciences, Universidad de Santiago de Chile, Usach. Santiago, Chile; Universitat Pompeu Fabra, Spain

## Abstract

Neurogenesis continues in the adult brain and in the adult olfactory epithelium. The cytokine, leukaemia inhibitory factor and nitric oxide are both known to stimulate neuronal progenitor cell proliferation in the olfactory epithelium after injury. Our aim here was to determine whether these observations are independent, specifically, whether leukaemia inhibitory factor triggers neural precursor proliferation via the inducible nitric oxide synthase pathway. We evaluated the effects of leukaemia inhibitory factor on inducible form of nitric oxide synthase (iNOS) expression, and cell proliferation in olfactory epithelial cell cultures and olfactory neurosphere-derived cells. Leukaemia inhibitory factor induced expression of iNOS and increased cell proliferation. An iNOS inhibitor and an anti-leukaemia inhibitory factor receptor blocking antibody inhibited leukaemia inhibitory factor-induced cell proliferation, an effect that was reversed by a NO donor. Altogether, the results strongly suggest that leukaemia inhibitory factor induces iNOS expression, increasing nitric oxide levels, to stimulate proliferation of olfactory neural precursor cells. This finding sheds light on neuronal regeneration occurring after injury of the olfactory epithelium.

## Introduction

Neurogenesis in the adult nervous system is limited to in a few areas of the brain [1] and to the olfactory epithelium [2], [3]. Adult neurogenesis is regulated by a variety of neurotrophins [4] and neuropoietic cytokines [5], [6]. The leukaemia inhibitory factor (LIF), a member of the gp130 family of neuropoietic cytokines, was originally identified as a macrophage proliferation and differentiation regulating factor [7], but several effects of LIF have been recently determined in neurogenesis. LIF signaling promotes the maintenance and self-renewal of mouse embryonic neural stem cells (NSCs) *in vitro* [8], [9], [10]. LIF expression is very low under normal physiological conditions, but it rapidly and transiently increases after several kinds of injuries, including trauma, seizure and ischaemia [11], [12], [13], [14]. The evidence suggests that LIF is involved in the recruitment of neural stem cells and progenitor cells after injury, which is likely to be the first step towards regeneration. LIF is also involved in neurogenesis in the olfactory epithelium, where it stimulates the proliferation of neuronal precursor cells [15]. The LIF receptor (LIFR) is expressed by neuronal precursor cells, neuronal axons and infiltrated macrophages during injury-induced olfactory epithelium regeneration [16], [17].

The continuous neurogenesis in the olfactory epithelium throughout the whole lifespan and its accessibility to experimental manipulation makes it a convenient model system to study the molecular factors that regulate this phenomenon [18], [19]. Neurogenesis gives rise to immature olfactory sensory neurons from mitotically active basal cells [2], [20], [21]. The globose basal cells are the major proliferating cell population in this tissue [22] and comprise multipotent progenitors that give rise to neurons, supporting cells and gland cells [20], [23], [24]. Another proliferating cell and putative neural stem cell is the horizontal basal stem cell that lies adjacent to the basement membrane, below the globose basal cells [25], [26].

Previously, we demonstrated that nitric oxide (NO) levels regulate cell proliferation and neuronal differentiation in adult olfactory epithelial cell cultures. Inhibition of the enzyme nitric oxide synthase (NOS) reduced proliferation of neuronal progenitors and stimulated their differentiation into neurons [18]. Release of NO had the opposite effect [18]. These observations suggest that NOS activity and/or expression may be induced in olfactory epithelium during regeneration, stimulating neuronal precursor proliferation [18]. This effect appears to involve the inducible (iNOS) and the neuronal (nNOS) isoforms of this enzyme, but the underlying mechanism is unknown. The mitogenic action of NO as a mechanism of injury-induced regeneration has been previously reported in other areas of the nervous system [27]. Furthermore, NO donors can assist in the recovery after brain injury, partly by increasing neurogenesis in the dentate gyrus of the hippocampus and the subventricular zone [28], [29], [30], [31].

In this study we investigated whether the proliferative effect of LIF acts via induction of iNOS expression in neuronal precursor cells, with the subsequent rise in NO, promoting the proliferation of these cells. This idea is supported by evidence that during injury-induced neuronal regeneration there is increased expression of both LIF [12], [14] and NOS [29], [32], [33]. Furthermore, activation of JAK/STAT signalling pathway induces iNOS expression in the immune system [34], [35] and LIF stimulation of neuronal precursor proliferation in the olfactory epithelium, acts via activation of JAK/STAT3 [36]. These observations strongly suggest that LIF-stimulation of olfactory precursor cell proliferation might act by inducing iNOS expression and subsequent activity.

We assessed this hypothesis using two cellular models: primary cultures of olfactory epithelium cells and olfactory neurosphere-derived cell cultures. Primary cultures of olfactory epithelial cells comprise a mixture of different cell types, including horizontal basal cells, the putative stem cells [25], [37] and globose basal cells, putative stem-like neuronal precursors [18], [19] that give origin to olfactory neurons and supporting cells. Neural stem cells are grown *in vitro* in “neurospheres” which contain stem cells, neural progenitors and developing neurons and glia [20], [38]. Neurospheres from olfactory mucosa are multipotent [20] and provide a source of regenerating cells to study neurogenesis [39].

In this work we show that LIF induces iNOS, which in turn promotes neuronal precursor proliferation. Although LIF and NO have been previously implicated in neurogenesis, promoting cell proliferation of embryonic and adult neuronal precursors, this is the first report of a common pathway linking these mitogens in neural progenitor proliferation. The results presented here offer a plausible mechanism for injured neuronal tissue repair and the identification of some of the factors implicated in this process.

## Materials and Methods

### Ethical Statement

All animal work was conducted according to the guidelines and approval of the Animal Ethics Committee at Universidad de Chile, Santiago, Chile.

### Primary Cultures of Olfactory Neuronal Precursor Cells and Neurosphere Cultures in Adults Rats

Adult, outbred Sprague–Dawley rats weighing approximately 300 g were obtained from the Animal House (Faculty of Biological Sciences, Catholic University, Santiago, Chile). Animals were sacrificed by decapitation after being deeply anaesthetized with sodium pentobarbital (100 mg/kg). Olfactory epithelium primary culture was performed as previously described [18], [19]. Dissociated olfactory epithelial cells were plated on plastic 4×1.9 cm^2^ well culture dishes (Nunc), previously coated with 5 µg/cm^2^ human collagen IV (Sigma Chemical Co.) at a density of approximately 350,000 cells per well in 500 µL of serum-free DMEM/F12 culture medium (low-glucose, with L-glutamine, Gibco-BRL), ITS supplement medium (insulin–transferrin–selenium, Gibco-BRL) and Penicillin–Streptomycin (100 U/mL–0.1 g/mL, Sigma Chemicals Co.). To stimulate proliferation of non-neuronal cell types, the cultures were treated for 5 days with human recombinant epidermal growth factor (EGF; 25 ng/mL; Sigma Chemical Co.). Neurosphere cultures were prepared following the protocol of Murrell et al [20], with some modifications. Briefly, the olfactory mucosa was removed from the nasal septum, immediately placed in Hanks’ balanced salt solution (HBSS, Gibco-BRL) and incubated for 45 min at 37°C in 2.4 U/mL Dispase II (Boehringer, Mannheim). Olfactory epithelia were carefully separated from the lamina propria by dissection. The lamina propria was incubated with 1 ng/mL collagenase 1(Sigma Chem) during 10 min and gently triturated by passing cell clumps about 20 times through a micropipette to dissociate the cells. Olfactory epithelia were treated the same way, but without the enzyme treatment. The resulting cell suspension was transferred to a 15 mL conical centrifuge tube containing HBSS and centrifuged at 200 g for 5 min. The pellets from both tissues were resuspended together in DMEM/F12 culture medium (low-glucose, with L-glutamine, Gibco-BRL), containing 10% foetal calf serum (FCS) plus penicillin/streptomycin 1X (Sigma Chem). Cells were plated on 35 mm plastic well culture dishes (Nunc, Co) at a density of 350,000 cells per well in 2 mL of medium. Cells were grown to confluence and plated into flasks of sequentially increasing sizes. Cells were then transferred to plates pre-treated with poly-L-lysine (1 µg/cm^2^; Sigma) at a density of 400,000 cells per well in 2 mL of DMEM/F12 medium, with ITS supplement medium (Gibco-BRL) and Penicillin–Streptomycin (100 U/mL–0.1 g/mL, Sigma Chemicals Co.), supplemented with 50 ng/mL EGF and 25 ng/mL FGF-2 (Calbiochem). Under these conditions, these cells grow into spherical forms called neurospheres after a week in culture. The neurospheres were selected by size (∼100 µm), harvested individually using a 200 µL micropipette and dissociated using Trypl E (Gibco) and re-cultured under the same conditions; this full procedure was repeated at least three times in order to avoid contamination with other cell types. Third or subsequent generations of neurospheres of ∼100 µm were individually collected and dissociated with Trypl E for subsequent experiments. Same generation neurospheres were used in all the experiments.

### RNA Isolation and RT-PCR

Cells were harvested by scraping and washed once in PBS. Total RNA was isolated by using the RNeasy mini Kit (Qiagen, Valencia, CA). Contamination with genomic DNA was prevented by a DNase1 treatment (Qiagen, Valencia, CA). mRNA was reverse-transcribed and cDNA using M-MLV reverse transcriptase (5 U/µL, Promega) with 1 hour of incubation at 42°C using a thermocycler 2027 Gradient Thermal Cycler. For the PCR where used Taq DNA polymerase (5 U/µL, Invitrogen). The primers used and the PCR conditions were the following: Inducible nitric oxide synthase (iNOS, Nos2) forward 5′- CTTTCTGGCAGCAGCGGCTC 3′, reverse 5′-GCTCCTCGTAAGTTCAGC 3′ (annealing T° 65°C) and Tyrosine 3-monooxygenase/tryptophan 5-monooxygenase activation protein, zeta polypeptide. (YWHAZ): forward 5′- GTCATCTTGGAGGGTCGTCT 3′, reverse 5′- GCTTCTTGGTATGCTTGCTGT 3′ (annealing T°: 55°C).

All cDNA reactions were subjected to an initial 5 min denaturing cycle at 94°C and to a final elongation step of 10 min at 72°C. The final concentrations of the PCR reagents were as follows: 1x PCR buffer, 1.5 mM MgCl_2_, 0.2 mM dNTP mix, 0.5 mM of both forward and reverse primers, 1 U Taq DNA polymerase (Invitrogen). PCR products were electrophoresed through a 2% agarose gel and visualized by ethidium bromide staining. Data were expressed as % relative to control medium and normalized by expression of housekeeping gene YWHAZ.

### Real Time PCR (qPCR) Protocol

qPCR was performed using Maxima SYBR Green/Fluorescein qPCR Master Mix(2X) (Fermentas), following the kit protocol directions with 40 amplification cycles at 60°C of annealing temperature with one cycle of dissociation curve from 55°C to 95°C. The primers used were the same as above. For analysis, the curve threshold (Ct) and the following parameters were used; ΔCt: Ct of each reaction subtracted to Ct house keeping gene YWHAZ. ΔCt constant: Averages of controls Ct subtracted to average housekeeping YWHAZ. ΔΔ Ct: ΔCt subtracted to ΔCt constant. Finally, for calculate the target genes expression we used the formula 2 ^(-ΔΔ Ct)^. Data were expressed as mean of expression increment fold ± SEM.

### Immunofluorescence

Cultures grown in glass coverslips covered with collagen IV were fixed with 4% paraformaldehyde and washed with phosphate-buffered saline (PBS, pH 7.2). Immunofluorescence was performed for LIFR, (1∶400, RD-System, monoclonal antibody, mouse); and iNOS (1∶500, Sigma Chemical Co, polyclonal antibody, rabbit). Non-specific staining was blocked with non-immune serum, appropriate for the secondary antibody, at a dilution of 1∶10 in PBS containing 5% non-fat powder milk, 4% bovine serum albumin (Sigma Chemical Co.) and 0.1% Triton X-100, for 1 hour at room temperature. The primary antibodies were incubated overnight at 4°C, followed by three 5 minutes washes and the incubation with the appropriate secondary antibody conjugated with the fluorescent agents Alexa 488 or 555 (1∶500, Molecular Probes) for 1 hour at room temperature. Finally, the nuclei were stained with 4',6-diamidino-2-phenylindole dihydrochloride (DAPI) (1∶1000) for 10 min.

For BrdU staining, cells were treated for 30 min with 2 N HCl at 60°C after fixation, followed by three 5 minutes washes with 0.1 M Borate buffer, pH 8.5. Non-specific staining was blocked with non-immune serum, appropriate for the secondary antibody, at a dilution of 1∶10 in PBS, followed by three 5 minutes washes, the antibody against BrdU (Dako, monoclonal) was used at a dilution of 1∶100 at 4°C and incubated overnight. After three washed of 5 minutes, the secondary antibody conjugated with the fluorescent agent Alexa 488 (1∶500, Molecular Probes) was incubated for 1 hour at room temperature. Finally, the nuclei were stained with DAPI (1∶1000) for 10 min. Immunofluorescence quantification was performed using the Image J software. Five different regions of interest were defined and half the intensity fluorescence was determined and normalized respect to the control group for three independent experiments, data were expressed as mean ± SEM.

Data were subjected to non-parametric Mann-Whitney test with α = 0.05, (GraphPad Prism 4.0, GraphPad Software, San Diego, CA, USA).

### Flow Cytometric Analysis

The flow cytometric analysis for expression of the proteins in study was performed on dissociated neurosphere-derived cells (∼200.000 cells for each experimental group). These cells were centrifuged for 5 minutes at 200 g, the pellet was resuspended in 4% PFA and fixed for 15 min. Cells were spun down for 5 minutes at 200 g, washed three times with PBS and incubated for 1 hour with blocking solution as described above in the immunofluorescence section. The primary antibodies against iNOS and LIFR were graduated previously and used in a dilution of 1∶200, the cellular pellet were incubated with each antibody overnight at 4°C with gentle agitation. After three 5 minutes washes with PBS, the samples were incubated with the corresponding secondary fluorescent antibody previously titrated and incubated for 2 hours at room temperature, finally the samples were washed with PBS and placed in PBS +2% FCS for the flow cytometric analysis. The controls for all experiment were an auto-immunofluorescence (without any antibodies) and a negative control (no primary antibody). The number of positive cells and fluorescence intensity was determined using a flow cytometer (BD FACS Canto II), with the emission at 488 nm for all markers. All data was analyzed using FACS DivaTM software (Becton Dickinson). Data represent the average of three independent experiments ± SEM. The intensity of fluorescence was normalized with respect to the fluorescence obtained from control basal minimal medium, performed independently on three different aliquots of cells. Data were subjected to non-parametric Mann-Whitney test with α = 0.05, (GraphPad Prism 4.0, GraphPad Software, San Diego, CA, USA).

### Expression of iNOS and LIFR Induced by LIF

Cells derived from neurospheres were plated on plastic 6×9.6 cm^2^ well culture dishes (Nunc) or on glass coverslips included in the wells, previously coated with 5.l µg/cm^2^ human collagen IV (Sigma Chemical Co.) and dried overnight at room temperature under sterile conditions. Cells were dissociated with Trypl E and plated at a density of 400,000 cells per well in 2 mL DMEM/F-12+10% FCS medium for 24 h, followed by a 24 h, the cells were wash with PBS 1x and treated with three different media: control medium (DMEM/F-12+ ITS), as negative control, EGF medium (DMEM/F-12+ ITS +25 ng/mL EGF, Calbiochem), as positive proliferation control, and LIF medium (DMEM/F-12+ ITS +20 ng/mL LIF, Chemicon). The samples were analysis for RT-PCR, immunofluorescence and flow cytometry.

In order to determine the temporal course of LIF-induced iNOS expression, cells were grown in DMEM/F-12+10% FCS to 70% confluence, and incubated with LIF medium for 0, 6, 12 and 24 hours. Data were subjected to non-parametric one-way variance analysis, two-tailed, with α = 0.05, followed by Bonferroni’s test (GraphPad Prism 4.0, GraphPad Software, San Diego, CA, USA).

### Analysis of Cell Proliferation

Cells derived from neurospheres were treated with control medium (DMEM/F-12+ ITS), as negative control, EGF medium (DMEM/F-12+ ITS +25 ng/mL EGF), as positive proliferation control, or LIF medium (DMEM/F-12+20 ng/mL LIF), in presence or absence of the iNOS blocker, L-NIL, the NO donor, SNAP or an anti-LIFR blocking antibody. In all the experiments L-NIL and SNAP were added at the same time as LIF treatment and the anti- LIFR blocking antibody was pre-incubated six hours before LIF treatment. Cell proliferation was quantified using the thymidine analogue, BrdU (2 mM; Sigma Chemical Co.). BrdU was added to the cultures for the last 6 h *in vitro* before fixation and immunochemistry was performed as described above. BrdU positive cells were counted and expressed as percentage of positive cells of the total cells ± SEM in a total of five independent experiments. Data were subjected to one-way variance analysis, two-tailed, with α = 0.05 (GraphPad Prism 4.0, GraphPad Software, San Diego, CA, USA).

## Results

### LIF Stimulated iNOS Expression and Cell Proliferation in Primary Cultures of Olfactory Cells

**Figure 1:**
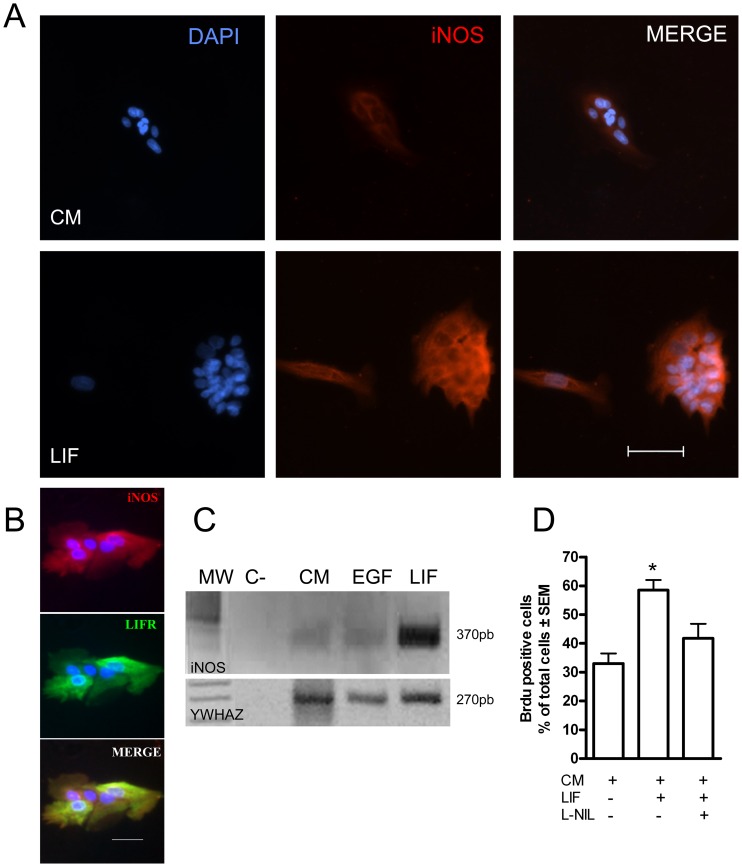
Primary cultures of olfactory epithelial cells expressed iNOS and LIFR. **A**. iNOS Immunofluorescence in cells grown for 24 h in control medium and in the same medium containing LIF. Blue: nuclei stained with DAPI. Red: Immunoreactivity to iNOS. Scale bar: 50 µm. **B**. Co-localization of iNOS (red) and LIFR (green) in cells grown in LIF. Blue: DAPI stained nuclei. Scale bar: 50 µm. **C**. Reverse transcriptase-polymerase chain reaction (RT-PCR) revealed mRNA for iNOS (upper panel) and YWHAZ, as a loading control (lower panel) in rat olfactory mucosal cells grown in control medium (CM), and in medium containing EGF, LIF and L-NIL. The negative control contained no mRNA. MW: DNA size markers; the predicted band for iNOS is 370 bp. **D**: Quantification of Brdu immunofluorescence of cell grown in control medium (CM), LIF containing medium and L-NIL containing medium. Data were expressed as mean of % Brdu positive cells ± SEM. *p<0.05 with respect to the control group.

In order to determine whether olfactory neuronal precursor cells are capable of responding to LIF by expressing iNOS, the expression of iNOS and LIFR was examined in primary cultures of olfactory epithelial cells; these cultures contained only neuronal precursor cells, except for a few supporting cells [19]. Neuronal precursor cells were grown in control medium or in the presence of LIF. A basal level of iNOS mRNA and protein expression was observed in cells grown in control medium, but it increased after 24 h of LIF treatment (Fig. 1A, C). Also, double immunolabeling using antibodies against iNOS and LIFR revealed co-expression of both proteins, suggesting that these cells respond to LIF by inducing iNOS expression (Fig. 1B).

The effect of LIF on cell proliferation was studied in primary cell cultures. Cells were grown in control medium or in the presence of LIF and the iNOS inhibitor L-NIL for 24 h. Minimum medium was used as negative control and EGF-containing medium (25 ng/mL) as positive control. The BrdU-positive cells were counted and are presented as percentage of the total number of cells (n = 4). These results indicate that LIF increased cell proliferation (Fig. 1D). The percentage of LIF-treated cells that incorporated BrdU (58.5±3.6%) was significantly larger than control cells in control medium (33.0±3.6%, Fig. 1D, p<0.05), an effect that was reduced by the iNOS inhibitor, L-NIL (Fig. 1D).

### LIF Stimulated iNOS Expression in Olfactory Neurosphere-derived Cells

**Figure 2:**
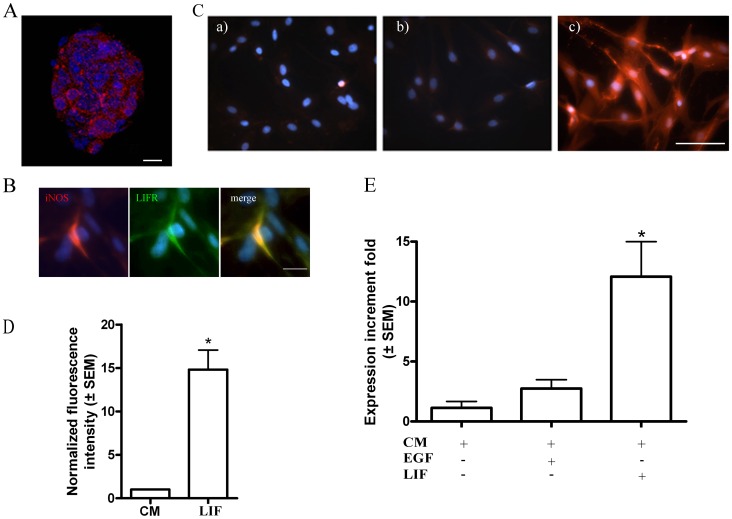
Olfactory neurosphere-derived cells expressed LIF. **A.** iNOS immunofluorescence (red) in a neurosphere. Scale bar: 20 µm. **B**. Co-localization of LIFR (green) and iNOS (red) in cells treated with LIF. DAPI-stained nuclei are blue. Bar: 25 µm. **C**. Immunofluorescence for iNOS in cells grown in control medium (a), EGF-containing medium (b) and LIF-containing medium (c). Scale bar: 50 µm. **D**. Flow cytometric analysis showing that LIF increased the iNOS expression measured by the mean intensity of fluorescence of the cells in each group. * p<0.05 respect to the control groups. **E**. qPCR revealed iNOS mRNA in rat olfactory neurosphere cells grown in control medium (CM), EGF-containing medium and LIF containing medium expressed as the mean ± SEM of the iNOS expression increment fold respect to the control group (CM). *p<0.05 respect to the control groups.

Subsequent experiments were undertaken on neurospheres generated from olfactory mucosa [20], because they offer a better defined neuronal stem cell/precursor culture, which can be propagated for multiple passages. Basal iNOS expression was observed by immunofluorescence in neurospheres (Fig. 2A). After the neurospheres were dissociated and maintained in different culture media for 24 h, LIF-treated cells co-expressed LIFR and iNOS (Fig. 2B), suggesting the involvement of LIF on iNOS expression. In order to evaluate this further, cells were grown for 24 h in different media and the cultures analysed for iNOS protein expression by immunofluorescence (Fig. 2C) and by flow cytometry, (Fig. 2D; n = 3) and mRNA expression (by q-PCR, Fig. 2E. n = 3). LIF treatment significantly induced the expression of iNOS mRNA and protein, compared with control groups. Quantification of the iNOS mRNA expression indicated that it was significantly increased in 12.08±2.9 fold after LIF treatment with respect to the control and EGF-containing medium (1.33±0.5 and 2.8±0.7 fold, respectively) (Fig. 2E, p<0.05). Although a similar proportion of cells expressed iNOS (Control: 79.1±0.1%; n = 10.2±8 cells; LIF-treated: 82.8±4.5%; n = 10,130±66 cells), there was a 14-fold increase in fluorescence intensity of in cells grown in LIF compared to control medium (Fig. 2D, p<0.05), indicative of increased iNOS expression.

**Figure 3:**
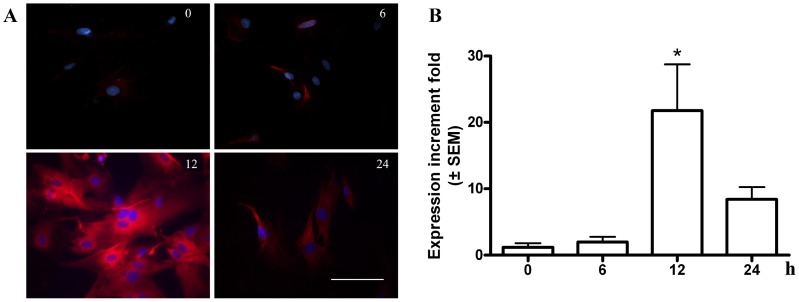
Temporal course of LIF-induced iNOS expression. **A.** Immunofluorescence of iNOS in cells treated with LIF for 0 (a), 6 (b), 12 (c) and 24 h (d). Blue: DAPI nuclear staining. Scale bar: 50 µm. **B.** qPCR iNOS mRNA treated as in A of three independent experiments expressed as the mean ± SEM of the iNOS expression increment fold respect to the control group (CM). * p<0.05.

In order to determine the temporal course of LIF-induced iNOS expression, cells were harvested at 0, 6, 12 and 24 h after LIF treatment and the iNOS expression was measured by immunofluorescence and q-PCR. iNOS immunofluorescence increased with time, reaching a peak at 12 h of LIF treatment (Fig. 3A; n = 3). iNOS mRNA was also induced by LIF treatment, reaching a maximum after 12 h (21.8±6.8 fold) compared with the expression at time 0; (1.2±0.6; at 6 h, 1.9±0.8 and 8.4±1.8 fold at 24 h; n = 3; Fig. 3B).

### LIFR Antibody Blocked LIF-induced iNOS Expression

**Figure 4:**
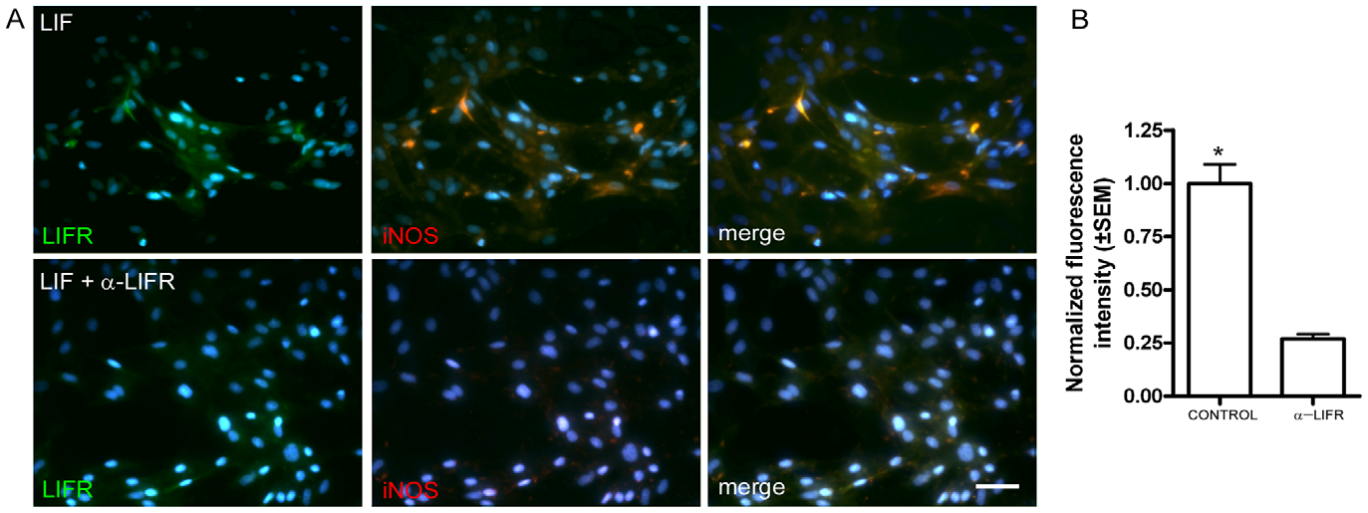
LIF-induced expression of iNOS expression was LIFR-dependent. **A.** Cells treated with LIF in presence and absence of a blocking antibody to LIFR which reduced immunofluorescence for LIFR (green) and iNOS (red). Blue: nuclear staining with DAPI. Scale bar: 50 µm. **B**.-Quantification of the average iNOS fluorescence intensity shown in A normalized respect to the control group in three independent experiments. * p<0.05.

Cells were treated with LIF in the presence and absence of the LIFR blocking antibody, and iNOS expression was determined by immunofluorescence. Neurosphere-derived cells were incubated in medium supplemented with LIFR blocking antibody (40 ng/mL) 6 h prior treating them with LIF for 24 h. Control cells were treated for 24 h with LIF without the blocking antibody. In the LIF-treated cultures, iNOS and LIFR expression was induced. In contrast, the antibody strongly reduced LIF-induced iNOS expression (Fig. 4 A–B; n = 3).

### LIF-induced Cell Proliferation is iNOS-dependent

**Figure 5:**
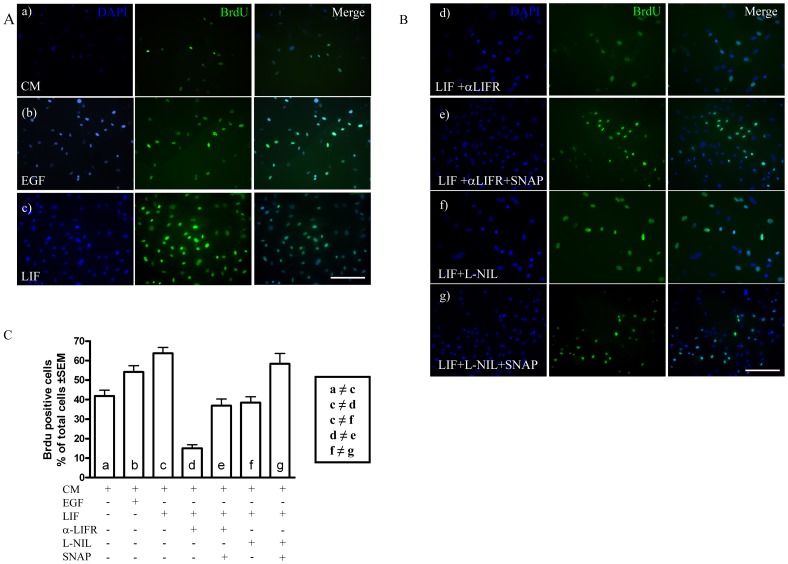
LIF-induced proliferation depends on iNOS. **A**. Immunofluorescence for BrdU (green) in cells grown in control medium (CM, a), EGF-containing medium (b) and LIF-containing medium (c). Blue: nuclear staining with DAPI. Scale bar:100 µm. **B**. BrdU immunofluorescence (green) in the presence of the LIFR blocking antibody (d), the LIFR blocking antibody plus the NO donor SNAP (e), the iNOS inhibitor L-NIL (f) and L-NIL plus SNAP (g). Blue, nuclear staining with DAPI. Scale bar: 100 µm. **C.** Quantification of data shown in A and B. ≠ * p<0.05 between the groups.

In order to study whether iNOS is a downstream element in the LIF-induced cell proliferation pathway, proliferation was measured by incorporation of BrdU in LIF-treated cells (20 ng/mL) in the presence or absence of the LIFR blocking antibody, with the iNOS inhibitor, L-NIL or the NO donor, SNAP for 24 h, alone or combined (Fig. 5). Minimum medium was used as a negative control and EGF-containing medium (25 ng/mL) as a positive control. The BrdU-positive cells were counted and presented as percentage of the total number of cells (n = 9 experiments). The results indicate that LIF increased cell proliferation (Fig. 5A). The percentage of LIF-treated cells incorporating BrdU (63.8±3.2%) was significantly larger than control cells in control medium (41.8±3.0%, Fig. 5C, p<0.05), an effect that was prevented by the anti-LIFR antibody (Fig .5B–d) and by L-NIL (Fig. 5B–f). The incubation with LIFR blocking antibody reduced cell proliferation to 15.1±2.9% (Fig. 5C, p<0.05), showing that besides blocking the effect of added LIF, the antibody blocked a basal LIF activity in the control condition. When iNOS activity was inhibited with L-NIL, the observed LIF-induced cell proliferation was significantly reduced (38.4±3.1%, p<0.05, Fig. 5C), demonstrating that the LIF effect was iNOS-dependent. Cell proliferation partially recovered when NO was generated independently of iNOS, using SNAP, in cell cultures treated with the anti-LIF blocking antibody (Fig. 5B–e, p<0.05) and in cell cultures treated with L-NIL (Fig. 5-B–g, p<0.05). The proportion of BrdU-positive cells rose with respect to the cells treated with LIF and LIFR antibody (58.3±5.4 and 36.9±3.4%, respectively; p<0.05; Fig. 5C).

## Discussion

The present study demonstrates the capability of olfactory neural progenitors to respond to LIF (they expressed LIFR mRNA and protein) and that the same cells that expressed LIFR also expressed iNOS. LIF treatment increased iNOS expression and cell proliferation. The effects of LIF on cell proliferation were prevented by inhibiting iNOS and by blocking the LIFR, while an NO donor had the opposite effect. These results demonstrate that iNOS and NO production are downstream elements of the LIFR signaling pathway in the stimulation of neuronal progenitor proliferation. The proliferative effect of NO [18], [29], [30], [40], [41] and LIF [9], [10], [42], [43] have been independently reported in the nervous system, including olfactory epithelium, but the connection between these pathways had not been previously described. With NO downstream of LIF, the potential is there to amplify spatially the effects of LIF signalling on individual cells to their surrounding neighbouring cells, based on the fast diffusion of NO.

When LIF binds to LIFR, the JAK/STAT pathway is activated, inducing the expression of several target genes, including the gene encoding iNOS. For example, the human iNOS gene has a STAT1 nuclear protein binding site in its promoter region [44] **.** In fact, the proliferative action of LIF in the olfactory epithelium is mediated by STAT3 [36] and in some cells acts on iNOS expression by binding to NFkB [45]. LIF induces iNOS mRNA expression in syncytiotrophoblast cells [46] and the iNOS activity and protein expression up-regulation in response to LIF has been previously reported in vascular smooth muscle [47], [48]. In the present study iNOS was expressed at basal levels by the same cells that expressed LIFR, but the characterization of the LIF transduction pathway that leads to iNOS expression is beyond the scope of the present study. Interestingly, the STATs and NF-κB cooperation through the polymerase II promoter recruitment and the phosphorylation of its carboxy terminal domain, respectively, has been reported as a prerequisite for the elongation of iNOS mRNA in macrophages [49].

Nitric oxide is a gaseous second messenger that has been implicated in neurogenesis promoting both, cell proliferation and cell differentiation. In the nervous system, as in other tissues, there is evidence that NOS inhibition increases proliferation of mouse subventricular zone precursors in vitro [40] and *in vivo* [50], [51], whereas exogenous nitric oxide decreases the number of proliferating cells in the Xenopus tadpole brain [52]. In contrast to these observations and in agreement with our study, a nitric oxide donor increases cell proliferation in the dentate gyrus and subventricular zone in young adult rats during recovery from ischaemic stroke [31].

NO has been implicated in cell proliferation [18], [53], [54] as a quick and transient response to damage. The endothelial and neuronal isoforms of NOS produce a small and localized intracellular rise in NO; in contrast, iNOS produces a massive rise in NO after a cellular damage [18], [55]. Here we show that the iNOS expression induced by LIF (and the subsequent NO production) occurs in a rather fast and transient manner. This is compatible with the reported action of NOS during tissue regeneration, where a high and transient production of NO occurs, avoiding in this manner a larger tissue damage by oxidative stress [29]. This mechanism has been also described in the immune system, where a massive cellular injury triggers an inflammatory process; in this case, the damaged cells can produce and release cytokines that activate cell proliferation and tissue regeneration [56]. The response to LIF in the present study was rapid: an increase in the iNOS mRNA and protein expression was observed twelve hours after LIF treatment. This suggests that the LIF-iNOS pathway could be quickly activated, promoting the proliferation of neuronal stem cell/precursor cells and the renovation of the olfactory epithelium after injury. The lateral spread of NO from the LIF-stimulated cells would spatially amplify the proliferating signal to surrounding neuronal progenitors, ensuring a robust response to tissue damage.

We observed that LIF increased cell proliferation, in agreement with the effect of this cytokine previously reported in the olfactory epithelium [12], [43]. LIF-induced cell proliferation was significantly reduced by the anti-LIFR blocking antibody, confirming the specificity of the effect. Similarly, LIF-induced cell proliferation was suppressed by an iNOS inhibitor, an effect that was overcome by the addition of a NO donor to the medium. This agrees with the L-NIL antiproliferative and SNAP proliferative effects previously described by our group [18].

LIF and NO have been previously implicated in neurogenesis, promoting proliferation of embryonic and adult neuronal precursors, but a common pathway connecting these mitogens had not been previously described in neural progenitors, although a similar mechanism was reported in smooth muscle, where LIF induces a rise in NO, promoting cell proliferation [48]. In this paper we offer strong evidence that at least one of the mechanisms involves the induction of iNOS, with the subsequent intracellular NO increment inducing the neuronal precursor proliferation. Taken together, the results presented here show a mechanism participating in injured neuronal tissue repair and the identification of some of the factors involved in this process. This could be of importance for the design of future therapies for neuronal regeneration.
